# Mechanistic Basis of Branch-Site Selection in Filamentous Bacteria

**DOI:** 10.1371/journal.pcbi.1002423

**Published:** 2012-03-08

**Authors:** David M. Richards, Antje M. Hempel, Klas Flärdh, Mark J. Buttner, Martin Howard

**Affiliations:** 1 John Innes Centre, Norwich Research Park, Norwich, United Kingdom; 2 Department of Biology, Lund University, Lund, Sweden; University of Illinois at Urbana-Champaign, United States of America

## Abstract

Many filamentous organisms, such as fungi, grow by tip-extension and by forming new branches behind the tips. A similar growth mode occurs in filamentous bacteria, including the genus *Streptomyces*, although here our mechanistic understanding has been very limited. The *Streptomyces* protein DivIVA is a critical determinant of hyphal growth and localizes in foci at hyphal tips and sites of future branch development. However, how such foci form was previously unknown. Here, we show experimentally that DivIVA focus-formation involves a novel mechanism in which new DivIVA foci break off from existing tip-foci, bypassing the need for initial nucleation or *de novo* branch-site selection. We develop a mathematical model for DivIVA-dependent growth and branching, involving DivIVA focus-formation by tip-focus splitting, focus growth, and the initiation of new branches at a critical focus size. We quantitatively fit our model to the experimentally-measured tip-to-branch and branch-to-branch length distributions. The model predicts a particular bimodal tip-to-branch distribution results from tip-focus splitting, a prediction we confirm experimentally. Our work provides mechanistic understanding of a novel mode of hyphal growth regulation that may be widely employed.

## Author Summary

Amongst the great variety of shapes that organisms assume, many grow in a filamentous manner and develop at least partly into a network of branches. Examples include plant roots, fungi and some bacteria. Whereas the mechanisms of filamentous growth are partially understood in fungi, the same cannot be said in filamentous bacteria, where our knowledge of hyphal growth regulation is very limited. To rectify this we have studied the bacteria *Streptomyces*, which are an excellent model for all hyphal bacteria. The protein DivIVA is known to play a critical role in controlling filamentous growth in *Streptomyces*, forming large foci at branch tips and smaller foci that mark sites of future branch outgrowth. However, until now nothing was known about how these foci first appear. We have shown experimentally that new foci appear via a novel mechanism, whereby existing tip-foci split into two clusters. The larger cluster remains at the growing tip, while the smaller cluster fixes onto the adjacent lateral membrane, where it grows in size, eventually initiating a new branch. By mathematically modelling how DivIVA foci grow, we show how this one simple mechanism of focus formation can quantitatively capture the statistical properties of the entire hyphal branching network.

## Introduction

The ability to break symmetry and establish an axis of polarity is crucial for the function and development of almost all cell types. In bacteria, such symmetry-breaking is often mediated by cytoskeletal elements inside the cell that direct new cell wall synthesis. Many rod-shaped bacteria (including *Escherichia coli*, *Bacillus subtilis* and *Caulobacter crescentus*) grow solely through the isotropic insertion of new cell wall material throughout the length of the lateral walls [1], [2]. Here, cell wall growth is directed by MreB, the bacterial ortholog of eukaryotic actin [3]–[6], whereas cell division is mediated by the bacterial tubulin ortholog, FtsZ. In these rod-shaped bacteria, polarity systems are required to identify and differentiate cell poles that remain inert during cell elongation. However, many other organisms enlarge by hyphal growth, a strategy that has proved successful for the exploitation of soil and other environments. Hyphal growth has evolved independently in both eukaryotic and prokaryotic microbes, including fungi and Gram-positive bacteria of the genus *Streptomyces*. This mode of growth depends on pronounced cellular polarity and the specific localization of cell envelope assembly to one cell pole in order to achieve tip extension. New sites of growth arise by hyphal branching, which requires the re-orientation of cellular polarity and the *de novo* establishment of new zones of cell wall synthesis from which lateral branches emerge. The result is a mycelial network in which the regulation of branching largely determines the morphology and behaviour of the mycelium as it spreads through the environment. However, the general principles that control such cellular branching have remained unknown. Here we report a novel mechanistic basis for branch-site selection in the mycelial actinomycete bacterium *Streptomyces coelicolor*. Since all hyphal bacteria are actinomycetes, this mechanism is likely to be widely relevant in this important phylum of bacteria, which account for the majority of commercial antibiotics.

Tip extension and hyphal branching in *Streptomyces* are independent of both MreB and FtsZ, and depend instead on the coiled-coil cytoskeletal-like protein DivIVA [7], [8]. A functional DivIVA-EGFP fusion localizes to tips and marks new branch points well before visible lateral outgrowth [9], [10]. Deletion of *divIVA* is lethal, whereas overexpression leads to greatly increased numbers of DivIVA foci along the lateral wall and *de novo* cell wall outgrowth at these foci [8]–[10]. These data suggest that DivIVA can direct cell polarity and recruit the machinery for cell wall synthesis. Additional cytoskeletal components may also be involved (for example, Scy [11]), together forming a tip-organizing complex. However, regardless of whether there are additional components, we can use DivIVA-EGFP as a marker to monitor the dynamics of the tip-organizing complex as a whole.

The branch-site selection mechanism that localises DivIVA to new sites along the lateral wall, from which branches subsequently emerge, was previously unknown. We therefore used the DivIVA-EGFP fusion to monitor the dynamics of the tip-organizing complex in *S. coelicolor* by live cell time-lapse imaging. These experiments revealed that the new DivIVA foci that initiate lateral branches arise predominantly by a novel tip focus-splitting mechanism that bypasses the necessity for initial nucleation or site-selection. In order to gain a deeper and more rigorous understanding of the regulation of hyphal branching, we then quantified hyphal branching patterns from still images, and developed a mathematical model of the DivIVA dynamics. As we will see, the model demonstrates that a remarkably simple tip-focus splitting mechanism is capable of quantitatively explaining all of our experimental branching pattern data, a result which is far from intuitive. Moreover, the model makes explicit predictions that we have experimentally verified. Intriguingly, a similar splitting mechanism has recently been reported in hyphal growth in fungi (*Neurospora crassa*) [12], raising the possibility that this simple mechanism may be widely applicable.

## Results

### Lateral DivIVA foci arise from splitting of apical foci

**Figure 1:**
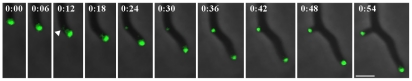
Evidence of tip-focus splitting, growth of foci and emergence of branches, in fluorescence-imaged *Streptomyces coelicolor* expressing *divIVA-egfp*. The tip always contains a large DivIVA focus and established tips extend at an approximately constant speed. At about 12 minutes, the DivIVA tip-focus undergoes splitting, leaving behind a new focus (arrow). As the tip continues to extend, the new focus remains in place on the membrane and grows in intensity. After about 42 minutes a new branch is formed at the position of the new focus, with the new focus now sitting at the tip of the new branch. Both the new branch and the original branch now continue to extend in length. Time in hours∶minutes. Scale bar: 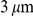.

Our previous studies have shown that DivIVA foci are always present at new branch points before outgrowth occurs [9], [10]. However, the origin of such DivIVA foci and the factors that determine their localisation have remained unclear [8]. To further understand the branching process, we have therefore studied more carefully how such foci are formed and traced their origin from time-lapse images. These experiments revealed that new small foci often arise from existing DivIVA foci at hyphal tips, by a process where a small cluster of DivIVA separates from the tip-focus and is left on the membrane just behind the tip. An example is shown in Figure 1 (see Video S1 for a movie of this figure). At around 12–18 minutes the focus of DivIVA at the tip splits and leaves behind a small focus on the adjacent membrane. As the tip continues to extend, the new focus remains fixed in place on the membrane and grows in size and intensity. In between 42 and 48 minutes a new branch is formed at the position of the new focus. Tip-focus splitting is only seen to occur from foci associated with extending tips; foci which have not yet initiated a branch, such as the smaller focus between 12 and 36 minutes in Figure 1, do not undergo splitting. We traced the origin of 52 nascent branches in time-lapse images and found that 42 of them (81%) were accounted for by tip-focus splitting events. Since only sufficiently large and intense DivIVA-EGFP foci are visible above the background fluorescence, some foci cannot be traced to their point of creation, and so this is likely to be an underestimate of the real proportion of branching arising from tip-focus splitting [10]. Thus, tip-focus splitting, rather than other potential mechanisms, such as spontaneous nucleation, appears to be the predominant method for focus initiation in wild-type cells.

### Measurements of hyphal growth and lateral branching

In order to quantitatively understand *Streptomyces* branch-site selection, we have measured two categories of distances from still images: the distance between the tip and the points where branches emerge, and the spacing between the branches themselves. Unlike the branch spacing, the tip-to-branch distance is not fixed: as the hyphae extend in length, the tip-to-branch distances increase. To avoid this difficulty we use our measurements to work out the tip-to-branch distance at the moment when the new branches appear, as discussed in *Materials and Methods*.

Unless care is taken when measuring the distributions from still images, it is easy to introduce biases that uncontrollably skew the data. For example, if only branching events relatively close to hyphal tips can be measured (as is inevitably the case for *Streptomyces* where individual hyphae cannot be traced into the dense mycelial clumps from which they emerge), then long branch-to-branch distances will never be recorded, even if they occur. As explained in *Materials and Methods*, we control for this effect by introducing a protocol so that all measured hyphae have effectively the same length, a distance we call the trim length. This is achieved by discarding hyphae which are shorter than the trim length and trimming those which are longer. This protocol does not eliminate measurement bias, but rather controls the bias so that our experimental measurements are unambiguous and can be precisely compared with data generated by our mathematical model (see below).

**Figure 2:**
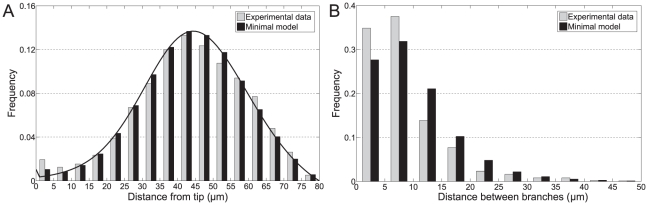
Comparison of histograms between minimal model and experimental data at 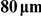 trim. (A) Tip-to-branch distribution. Analytic prediction is also shown (curved line). 1097 experimental data points. (B) Branch-to-branch distribution. 858 experimental data points.

The measured tip-to-branch and branch-to-branch distributions with a 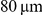 trim are shown in Figure 2. The tip-to-branch distribution has two distinct peaks, one between 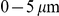 and one at 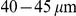 (Figure 2A). This might suggest that two distinct mechanisms are involved in producing new branches. Surprisingly, however, our later analysis will show that a single mechanism can account for both peaks.

### Minimal mathematical model of the growth of DivIVA foci

We assume that DivIVA foci, either on their own or as part of a tip-organizing complex, assemble the cell wall synthesis machinery to both extend hyphae and form new branches. Most new DivIVA foci do not immediately initiate a new branch (Figure 1). We assume this is a result of the small starting sizes of most foci. Foci must instead grow in size by accumulating DivIVA molecules from the cytoplasm until they contain enough molecules to initiate a new branch. To understand where new branches emerge we must therefore understand how the number of molecules, 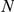, in a focus changes with time. We will refer to this number 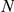 as the tip-focus size. We consider simple cooperative binding where the rate of DivIVA molecules joining a focus is linearly dependent on both the cytoplasmic DivIVA density, 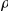, and the focus size, 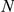 (alternative growth rules are considered in Supporting Text S1, but these alternatives give qualitatively similar results, with no better fit to the experimental data). Thus we have 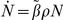, where 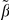 is a parameter independent of 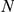 and 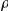. Although, in the minimal model, we assume foci never lose DivIVA molecules, including this process again makes little or no difference (see Supporting Text S1). We also assume that the cytoplasmic DivIVA density appearing in the above equation is the same for all foci (this assumption is justified by our full simulations, see Supporting Text S1). Thus we can replace 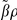 by the single parameter 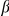, which we call the binding parameter, and consider 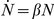. We assume that a focus starts with 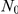 molecules and must reach 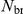 molecules before it can form a branch. We can easily solve the above equation for 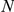 to find the time taken, 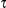, for this growth from 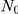 to 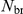. With an extension speed 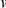 for established tips, the distance 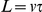 behind the tip where a branch appears is
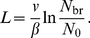
(1)


By comparing images like Figure 1 at 12 and 42 minutes, we estimate a typical value for 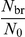 as between 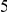 and 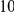, so that, to a rough approximation, 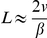. The absolute value of 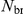 is difficult to determine, but since the fluorescence of a typical DivIVA focus is not dissimilar to that of an FtsZ ring, and since an FtsZ ring contains on the order of 10,000 molecules [13], we take 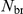 to be of a similar order of magnitude. The growth speed of an established tip, 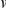, is measured from time lapse images to be about 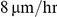. Due to the trimming issues discussed above, measuring a typical value for 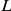 is not straightforward. In particular, using the average of a trimmed distribution, such as that in Figure 1A, will not give a good estimate. However, as explained in *Materials and Methods*, by studying the distributions over a range of trims, we estimate a value of about 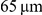 under the growth conditions used, which implies that 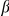 should be about 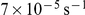. (See Figure S10 for a schematic of the colony morphology for different values of 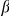.)

*Streptomyces* produces branches at a range of distances behind tips, producing a distribution of tip-to-branch distances. In our model, this is due to fluctuations in the parameters in Eq. (1). Note that, although we vary these parameters, we do not model the growth of foci themselves stochastically (instead using a deterministic differential equation) due to the large number (thousands) of molecules involved. Each binding event will itself be stochastic but the overall process involving many thousands of such binding events will be well described deterministically.

### The tip-focus splitting mechanism

So far we have been concerned with how the number of molecules in a pre-existing focus changes with time. We have not yet discussed the mechanism by which new foci are formed, the tip-focus splitting mechanism. Furthermore, after a tip-focus has undergone splitting, we are interested in the length of time before the focus can split again, which, after both foci have initiated new branches, will translate into the distance between branches. It is important to emphasise that, whereas the growth of foci controls the tip-to-branch distribution, it is the focus-splitting rules that control the branch-to-branch distribution.

The simplest assumption that could be made would be that the focus-splitting probability per unit time is constant, independent of when the tip-focus last split. This would describe a Poisson process and so imply an exponential distribution for the branch-to-branch distribution. However, as Figure 1B shows, for distances smaller than 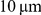 the branch-to-branch histogram is not described by a decaying exponential: these shorter distances are measured much less frequently than implied by a Poisson distribution.

This suppression of short branch-to-branch distances shows that focus-splitting events are not independent of each other: a tip-focus that has just split is less likely to immediately split again. One potential explanation is that the probability of tip-focus splitting depends on the tip-focus size, such that smaller tip-foci are less likely to split. For this reason we implement a minimum tip-focus size (a critical mass), 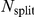, below which the tip-focus cannot split, with some constant focus-splitting probability per unit time, characterised by the parameter 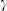, for all tip-foci above 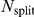. Splitting events cause the tip-focus to decrease in size and so, in some instances, such a splitting will cause the tip-focus size to drop below 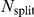. In that case, only after the tip-focus has absorbed more DivIVA from the cytoplasm will it have sufficient size to split again. This time delay effectively reduces the number of short branch-to-branch distances.

Although it is difficult to analyse tip-focus splitting analytically, it is useful to note that, in the limit where 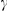 is very large (compared to 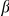), the branch-to-branch distance, 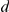, is given by
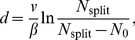
(2)a result which follows in a very similar way to Eq. (1).

### Fitting the minimal model

In order to compare the minimal model with the experimental data, we developed a simulation which grows *Streptomyces* hyphae, implements tip-focus splitting and focus growth, performs the trim to the required length, and extracts the distributions (see *Materials and Methods*). We used the parameters listed in Table 1 with 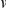, 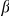, the mean initial focus size 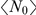, and the mean focus size for branch initiation 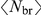 inferred from experiments (see above), and with the standard deviations in 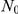 and 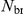, that is 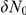 and 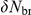, and 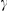 fitted to the experimentally determined tip-to-branch and branch-to-branch distributions at 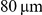 trim. We find that variations in just 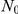 and 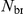 are sufficient to fit all the measured distributions. For simplicity we take 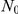 and 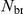 to follow independent truncated Gaussian distributions, where the truncation ensures that 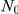 and 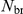 are always positive. This is required since Gaussian distributions assign non-zero probabilities to all values, whereas biologically foci cannot contain fewer than zero molecules. The means (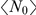 and 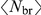) and standard deviations (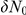 and 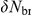) are those for the truncated distributions, rather than the full Gaussians. However, as shown in Supporting Text S1, other distributions do not qualitatively change our results.

**Table 1:** Main parameters and their values.

Parameter	Value
Tip growth speed, 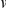	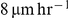
Binding parameter, 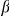	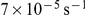
Mean initial focus size, 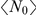	1,700
Standard deviation in initial focus size, 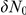	1,000
Mean focus size for branch initiation, 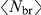	10,000
Standard deviation in focus size for branch initiation, 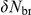	2,600
Minimum tip-focus size for tip-focus splitting, 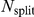	10,000
Tip-focus splitting probability per unit time, 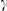	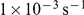

In our fitting, it was not immediately clear whether 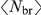 should be larger or smaller than 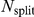. Note that although we allow the possibility that 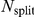 is less than 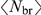 in the model, this does not mean that foci can split before they have initiated branches; DivIVA foci have only been observed to split when they are associated with a growing tip. However, 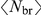 smaller than 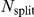 would imply that newly formed branches cannot normally produce their own branches until the tip-focus has grown further to size 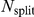. This in turn results in a gap between where a branch emerges from its parent hypha and the position of its first offshoot. We measured this distribution of distances and found no evidence for such a gap (see Supporting Text S1 and Figure S2), which implies that 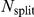 is equal to (or smaller than) 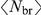. In our model we choose 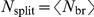, although smaller values of 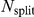 make little qualitative difference.

As shown in Figure 2, there is excellent agreement between the minimal model fits and the experimental data. For the trimmed tip-to-branch distributions, our model is sufficiently simple that this distribution can be calculated analytically (see Supporting Text S1) without recourse to simulations. The analytic prediction is also shown in Figure 2A and agrees extremely well with the simulation data, as expected. Note that the reason the tip-to-branch distribution drops to zero at 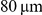 is a consequence of the trimming protocol rather than any inherent property of *Streptomyces*. We chose a 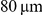 trim as a trade-off between distribution width and amount of data, but it is also possible to compare the model and the experimental data at other trims. Figures S8 and S9 show that there is also good agreement at trims of 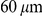 and 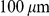.

We have checked that the tip-to-branch and branch-to-branch distributions generated by the minimal model are robust to changes in all the parameters in Table 1. Further, we tested that adding fluctuations in the tip growth speed, 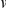, and the on-rate parameter, 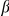, also do not qualitatively change these distributions (see Supporting Text S1). There is little to be gained by also considering fluctuations in 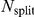 since the stochastic nature of tip-focus splitting is already included via 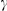, the tip-focus splitting parameter.

### Verifying a model prediction in the tip-to-branch distribution

**Figure 3:**
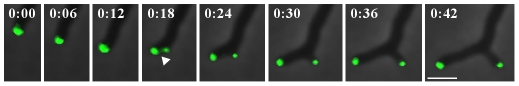
Example of branching at almost zero distance from the tip. The model indicates that this is due to tip-focus splitting events (arrow) where 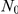 is greater than 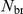. Time in hours∶minutes. Scale bar: 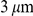.

**Figure 4:**
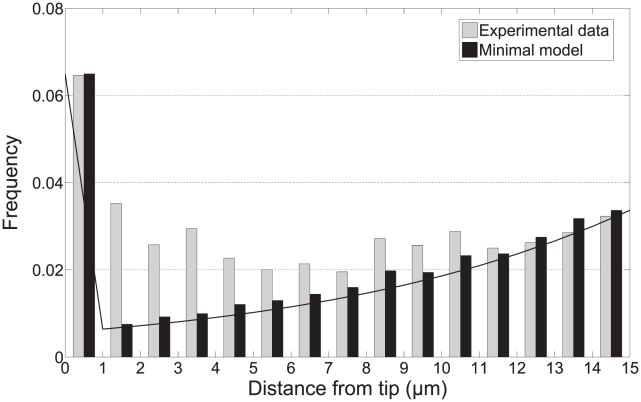
Comparison of tip-to-branch distribution at small distances between minimal model and experimental data at 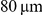 trim. Analytic prediction is also shown (curved line).

One of the most striking features of the experimentally measured tip-to-branch distribution, Figure 2A, is the peak at small distances. Naïvely it may be thought that a novel tip-focus splitting mechanism is required to account for this peak. However, our model predicts that this peak can be simply explained without additional assumptions. Since most new foci must attract more DivIVA molecules before they can initiate a new branch, the distributions of 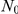 and 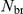 must be such that most new foci start with fewer than 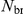 molecules. However, there is a small tail to the distributions that causes a few foci to have 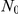 above 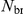, i.e. when they are formed these foci already have enough DivIVA molecules to initiate branch outgrowth. These foci will cause branching almost as soon as they are formed, very close to zero distance from the tip. We have directly observed such events and an example is shown in Figure 3 (see Video S2 for a movie of this figure). Furthermore, we also measured the total intensity of 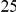 newly-produced foci from time-lapse images: 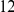 from cases where the new branch appears next to the tip and 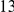 from normal tip-focus splitting events when the new branch appears much further back. In the first case the average intensity is almost three times greater than in the second case, supporting the hypothesis that events where the branch appears next to the tip correspond to the initial focus size, 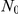, being much greater than average. The entire weight of the distribution with 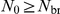 will give effectively zero tip-to-branch distances, which then naturally explains the peak at the origin in Figure 2A. Consequently, our model predicts that if the distribution is analysed with bins of smaller width, then the peak at the origin will become even more dramatic. After reanalysing the measured data, this prediction is strikingly confirmed, as shown in Figure 4. Although the peak in the 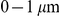 bin matches well, the agreement is not perfect in the range 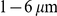. However, we believe this feature is an unavoidable artifact of how the data is analysed: the tip growth speed cannot be measured directly from still images, rather only the distribution of speeds is known, which necessarily slightly smears the data (see *Materials and Methods* and Supporting Text S1).

### Full model: curvature-dependent tip-focus splitting

It has been shown that the DivIVA orthologue in *B. subtilis* preferentially assembles on negatively-curved membranes, and this appears to be an important factor in targeting of the *B. subtilis* protein to cell poles and septation sites [14], [15]. Similarly, in *Streptomyces*, a preference for branches to emerge on the outer side of curved hyphae has been reported [10], which suggests, for example, that for tips that bend to the left, foci are more likely to form on the right inner membrane. Although the mechanism by which this occurs is not yet fully understood, it is possible to ask how such an effect impacts our model. To do so we developed and simulated a more detailed computational model (see Supporting Text S1), which implements hyphal growth in two-dimensional space. At each time step in the simulation, the direction of tip growth is randomly varied by a small amount, such that over sufficiently long distances (a few 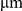), memory of the previous growth direction is lost. We postulate that tip-foci with sizes above 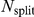 can split only when the local curvature near the tip is sufficiently high. Hence the earlier focus-splitting parameter, 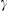, is understood as an effective parameter that can be replaced by growth direction variation and a curvature threshold. However, it is worth noting that if curvature is the origin of 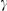, it must be quite a sensitive effect since during growth the mean curvature near the tip only changes by about 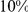. The full model (see Supporting Text S1 for full details and parameters) produces colony dynamics that match well with the wild-type phenotype (for example, see Videos S3 and S4). In particular, the tip-to-branch and branch-to-branch distributions are practically identical to the minimal model, thereby justifying our earlier simplifying assumptions.

### Under- and overexpression of *divIVA*

Since DivIVA is an essential protein, it cannot be completely removed. However, we can consider mild underexpression and various levels of overexpression. We first consider heavy overexpression. Previous work has examined hyphal morphology when *divIVA* was overexpressed in preformed hyphae to approximately twenty-five times its usual level [9], [10]. Such overexpression resulted in increased levels of cytoplasmic DivIVA, swollen hyphal tips and lateral hyperbranching. Interestingly, after inducing increased DivIVA production, many of the new branches developed well behind the tip positions at the moment of induction. This observation is unexpected since, in the minimal model, foci can only be produced from the splitting of tip-foci. It is possible that these new branches are due to foci that were already present at the time of induction but that were too small to be seen, and that overexpression subsequently caused them to develop into branches much more rapidly than normal. However, if this explanation were correct, wild-type *Streptomyces* would form many branches hundreds of microns behind the tips, a strategy which would be very inefficient in terms of nutrition acquisition. For this reason, we favour an alternative explanation, namely that these new branches arise from a separate mechanism of focus formation: spontaneous nucleation. In this process, due to the stochastic dynamics of molecules within the cytoplasm, occasionally a sufficient number of DivIVA molecules come together on the membrane and spontaneously form a cluster.

As is standard for nucleation dynamics [16], and as we confirmed by stochastic simulations, for cytoplasmic DivIVA densities below some threshold, the probability of spontaneous nucleation (involving the near simultaneous binding of multiple DivIVA molecules to overcome a nucleation barrier) is close to zero. Above this threshold, however, we find that the rate of nucleation rises approximately linearly with increasing cytoplasmic density. We assume that for the parameters chosen in Table 1, the DivIVA concentrations during wild-type growth fall well below this threshold and hence spontaneous nucleation does not occur. However, at 25-fold overexpression, this threshold is exceeded. In this latter case, we implemented spontaneous nucleation in our full model in the simplest possible way, by having a probability per unit length and time for spontaneously creating a new focus on the membrane, with a linear increase in nucleation probability with increasing cytoplasmic density above the threshold (see Supporting Text S1 for full details and parameters). We were then able to produce simulated colony dynamics which successfully matched the observed phenotype of 25-fold overexpression (for example, see Video S5).

In addition to heavy overexpression, we can also consider mild under- and overexpression. It was observed in [9] that underexpression seems to reduce the average tip-to-branch distance. It is important to realise that a change in DivIVA expression will probably not only affect the binding parameter 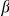 (since 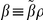, with 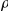 the cytoplasmic DivIVA density and 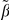 a constant), but also the tip growth speed 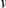. This is because DivIVA is a critical component of the tip-organizing complex, which is present at all growing tips, and which is presumably important for tip extension. Since 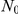 and 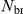 are unlikely to depend strongly on DivIVA levels, Eq. (1) shows that it is actually the ratio 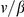 which controls the average tip-to-branch distance. When DivIVA is underexpressed it is likely that both 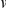 and 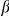 decrease. Since in this case the average tip-to-branch distance decreases, this result suggests that 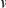 proportionally decreases by more than 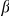. In the case of overexpression 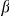 will increase. However, it is less likely that 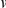 will also increase. This is because the tip-organizing complex, which is responsible for tip extension, is likely to consist of many components, of which DivIVA is only one. Unless other components in addition to DivIVA are overexpressed, the effect on tip growth speed could be small, with 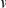 remaining approximately constant. Thus we predict that mild overexpression of DivIVA will reduce 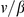 and so decrease the average tip-to-branch distance. If this is the case, then both mild under- and overexpression of DivIVA will reduce the average tip-to-branch distance, with wild-type levels corresponding to the longest tip-to-branch distance.

## Discussion

Streptomycetes, like other bacteria, lack the motor proteins, vesicle transport systems, and polarisome components that are fundamental in eukaryotic cell biology. Thus, tip extension in *Streptomyces* is likely to be simpler than in, for example, filamentous fungi. Given that a complex of polarity proteins (including DivIVA) must presumably first gather at future branch sites, understanding branch-site selection in filamentous bacteria involves understanding where, when and how these proteins cluster together in sufficiently large groups. One surprising feature of wild-type *Streptomyces* is that this clustering of polarity proteins is not a random, spontaneous process. Rather, we have shown that new branch sites are predominantly created from the tips of previous branches, by a tip-focus splitting mechanism.

One important question concerns the benefit of producing foci, and hence branches, by tip-focus splitting rather than spontaneous nucleation. One possibility is that this provides a more efficient method of acquiring nutrients. Spontaneous nucleation will produce new branches at positions well behind the tips. This outcome would be suboptimal since regions far behind the tips are likely to have already been well-exploited, with few remaining nutrients. Tip-focus splitting, on the other hand, only generates new foci at tips and so biases branching towards the growing ends of hyphae, where nutrients are still more plentiful. Another potential advantage is that tip-focus splitting allows for a greater level of control over exactly where branching occurs. Unlike spontaneous nucleation where branches can appear anywhere, tip-focus splitting produces branches with an average tip-to-branch distance determined by parameters such as the initial tip-focus size and the binding parameter. By modifying these parameters, it is possible to respond to external stimuli. For example, under conditions when branching further from the tip would be favourable, we speculate that this could be achieved by modifying DivIVA (or other proteins that affect its assembly) so that the binding parameter is decreased (this would correspond to a shift from the morphology shown in Figure S10B to that in Figure S10A).

The morphology of branching organisms can be characterized by both the distance from the tip that new branches appear and the inter-branch distance. Counter-intuitively, our model shows that these distances are controlled by rather different processes. The tip-to-branch distance is governed by how long it takes new foci to gather enough molecules to initiate a new branch. This is related to the initial focus size, 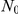, the size at which a new branch is initiated, 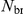, the tip growth speed, 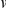, and the binding parameter, 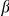. In contrast, the branch-to-branch distance is governed by how often foci are formed (how long foci take to develop into branches is now irrelevant). This is dependent on a partly overlapping, but nevertheless distinct set of parameters: the minimum tip-focus size for splitting, 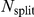, the initial focus size, 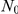, the tip growth speed, 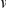, the binding parameter, 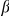, and the tip-focus splitting parameter, 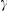.

We have focused on the control of branching during vegetative growth. However, there is a parallel question about how the first germ tube emerges from a spore. By imaging germinating spores expressing functional *divIVA-EGFP*, it has been shown that, exactly as in vegetative growth, a focus of DivIVA is first observed on the spore envelope, which then grows in size before initiating the first branch [9]. It is interesting to inquire how this first focus is formed. It is clear that the tip-focus splitting mechanism cannot be responsible since there are no previous DivIVA foci from which the first focus could arise. It is possible that other proteins, such as SsgA [17], aid DivIVA focus formation during spore germination. However, there is another possibility, that the spontaneous nucleation mechanism which plays a role when DivIVA is heavily overexpressed, is also responsible for the first DivIVA focus in a spore. If this is the case, then the DivIVA concentration within a spore would have to first rise high enough to overcome the nucleation barrier, an effect which may well be testable.

In fungi, branching also occurs at the cellular level and involves establishment of new cell poles at which apical growth will occur [18]. An apical cluster of vesicles and cytoskeletal elements named the Spitzenkörper has a prominent role in fungal tip extension. During branching, a new Spitzenkörper structure is established at the nascent branch tip, aided by proteins that direct cell polarity, cytoskeletal reorganisation, vesicle transport, and exo- and endocytosis (for reviews, see e.g. [18]–[21]). One of the components that appears to be involved in branch site selection prior to assembly of the Spitzenkörper structure is the protein complex termed the polarisome. Homologs of the budding yeast polarisome component Spa2p have been detected at hyphal tips in several fungi, and intriguingly, in *Neurospora crassa*, small foci of SPA-2-GFP were observed to detach from the major SPA-2 assemblies at elongating hyphal tips and subsequently give rise to new lateral branches [12]. This observation strongly suggests that, in addition to streptomycetes, tip-focus splitting mechanisms are also involved in the establishment of new hyphal branches in filamentous fungi.

Streptomycetes appear to regulate hyphal growth and branching in a simple way. Indeed, we have found that a remarkably simple model can quantitatively explain the statistical properties of the entire hyphal network. Even the bimodal nature of the tip-to-branch distribution originates from a single mechanism of forming new foci, combined with variation in the parameter values. It is tempting to speculate that tip-focus splitting might be used by many filamentous organisms amongst fungi and Actinobacteria. In fact, focus splitting could turn out to be a general mechanism in situations where discrete foci must be generated in a growing organism.

## Materials and Methods

### Strains, general methods and microscopy

*S.coelicolor* A3(2) strains M600 (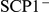 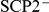), M145 (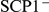 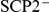) and K112 [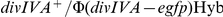], which produces DivIVA-EGFP, were pregerminated and cultivated at 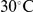 in YEME medium [22]. Hyphae were prepared for microscopy as described previously [9]. Samples were observed through a DIC 63× objective of a Nikon Eclipse 800 microscope equipped with a Pixera ProES600 camera and still images were taken with Pixera software and processed with ImageJ (National Institute of Health USA).

### Time-lapse imaging

Live cell time-lapse microscopy was performed essentially as described in [10]. In brief, hyphae of *S.coelicolor* strains were grown on 1% agarose pads with Oxoid antibiotic medium no. 3. Pads were sealed to the bottom by an oxygen-permeable Lumox Biofoil 25 membrane (Greiner Bio-One) and to the top by a coverslip. Samples were incubated at 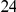 to 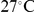 and observed using a Zeiss Axio Imager Z1 microscope, a 9100-02 EM-CCD camera (Hamamatsu Photonics), and Volocity 3DM software (Improvision). Images were captured every 6 minutes, processed by Volocity and analysed using ImageJ.

### Measurement of tip-to-branch distances

Still images do not normally capture the exact instant at which a new branch emerges. To find the tip-to-branch distance at the moment the branch emerged, we measure the length of the new branch, calculate how long it has been growing for, and determine where the tip was when the new branch emerged. The calculation incorporates an initial speed for new branch growth of about half that of established branches, increasing linearly in time until full speed is reached after about ninety minutes (see Figure S1). For details see the Supporting Text S1.

### Controlling for biases

When measuring tip-to-branch and branch-to-branch distances from still images, it is important to control biases that artificially skew the data. For example, as an extreme case, if the measured hyphae segments were all less than 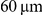 in length, it would then be impossible to measure any branch-to-branch distance greater than 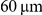. To control this problem we use the following protocol. Before any measurements are performed, all hyphae must be trimmed to some fixed length 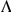: any hyphae shorter than this are discarded and, for those which are longer, only the segment within a distance 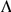 of the tip is included in the data set. The effect of trimming is to ensure that all measured hyphae are effectively of length 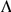. As a consequence, both the tip-to-branch and branch-to-branch distributions explicitly depend on the trimming length 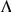.

### Estimation of average tip-to-branch distance

Estimating the average tip-to-branch distance from still images is complicated by the need to impose the trimming protocol on all measured data. The true average tip-to-branch distance is the average tip-to-branch distance at infinite trim. Distributions at progressively smaller trims have progressively smaller average tip-to-branch distances. The largest trim that we have a reasonable amount of data for is 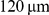, with an average tip-to-branch distance of 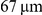. It is not obvious that this trim is sufficiently high to give a good estimate of the true average tip-to-branch distance. However, by fitting the full distributions at 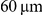, 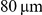 and 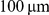 trims and extrapolating to infinite trim, this is seen to be a good approximation to the true average.

### Simulation details

We give details of the minimal model simulation here; details of the full model simulation can be found in Supporting Text S1. We simulate the growth of a single hypha starting with a single DivIVA focus at the tip (initially of size 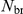) and keeping track of where branches appear. At each time step (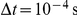), the hypha length is increased by 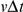, the tip-focus is increased in size according to 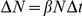, and the tip-focus splitting rules are implemented (i.e. a tip-focus above 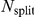 has a probability 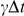 of splitting). If a new focus is created then its initial and final sizes, 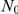 and 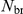, are chosen at random from truncated normal distributions, after which Eq. (1) gives the tip-to-branch distance. After the hypha has grown to sufficient length (we grow the hypha to twice the trim length in order to effectively randomise the initial conditions), the tip-to-branch and branch-to-branch distances are measured if they satisfy the trimming protocol with trim 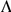, i.e. tip-to-branch distances are recorded only if the branch appears within a distance 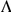 of the tip, and branch-to-branch distances are recorded only if both branches are within a distance 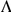 of the tip.

## Supporting Information

Figure S1 Tip growth speed against time in Oxoid antibiotic medium for an established hypha and a newly formed branch. Error bars show the standard error of the mean.
(EPS)

Figure S2Experimental distribution of distances from parent hypha to first offshoot at 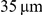 trim. 44 data points.(EPS)

Figure S3Comparison of model histograms at 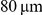 trim with 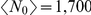 and 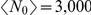. (A) Tip-to-branch distribution. (B) Branch-to-branch distribution.(EPS)

Figure S4Comparison of histograms at 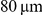 trim for linear growth model (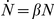, parameters in Table 1) and constant growth model (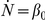, 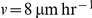, 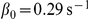, 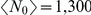, 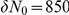, 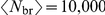, 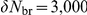, 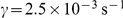, 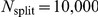). (A) Tip-to-branch distribution. (B) Branch-to-branch distribution.(EPS)

Figure S5 Analytic tip-to-branch distribution with infinite trim. This represents the “true” underlying distribution which can never be directly measured experimentally.
(EPS)

Figure S6Requirement for a branch to be included in the data set. (A) A growing branch which will be measured when it has grown another 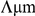. (B) A new focus is created at distance 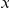 from the base. (C) This focus develops into a branch after the tip has grown a further 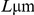, i.e. this branch has a tip-to-branch distance of 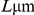. (D) Only branches within 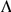 of the tip are used to collect data. So this branch will only be recorded if 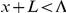.(EPS)

Figure S7Behaviour of the mode of the tip-to-branch distance distribution as a function of various model parameters, for both an infinite trim (blue line) and an 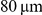 trim (red line). The infinite trim line is always higher than the 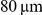 trim line. The black dotted line shows the wild-type parameter value. (A) As a function of the binding parameter, 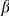. (B) As a function of the mean initial focus size, 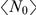. (C) As a function of the mean focus size for branch initiation, 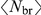.(EPS)

Figure S8Comparison of distributions between the minimal model and experimental data at 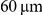 trim. Analytic tip-to-branch distribution is also shown (curved line). (A) Tip-to-branch distribution. 1876 experimental data points. (B) Zoomed tip-to-branch distribution. (C) Branch-to-branch distribution. 1215 experimental data points.(EPS)

Figure S9Comparison of distributions between the minimal model and experimental data at 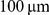 trim. Analytic tip-to-branch distribution is also shown (curved line). (A) Tip-to-branch distribution. 297 experimental data points. (B) Zoomed tip-to-branch distribution. (C) Branch-to-branch distribution. 257 experimental data points.(EPS)

Figure S10Schematic of colony morphology for various values of the binding parameter, 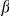. Red dots represent DivIVA foci. (A) Small value of 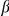. (B) Wild-type value of 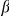. (C) Large value of 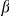.(EPS)

Text S1 Supporting text.
(PDF)

Video S1 Movie version of Figure 1. Evidence of tip-focus splitting, growth of foci and emergence of branches, in fluorescence-imaged *Streptomyces coelicolor* expressing *divIVA-egfp*. Time in hours∶minutes∶seconds.
(MOV)

Video S2 Movie version of Figure 3. Example of branching at almost zero distance from the tip. Time in hours∶minutes∶seconds.
(MOV)

Video S3 Example of the full model simulation output, showing *Streptomyces* starting from a spore and growing for about fourteen hours. Hyphae in green; DivIVA foci in red.
(GIF)

Video S4 Large-scale example of the full model simulation output, showing *Streptomyces* starting from a spore and growing for about eleven hours. Hyphae in green; DivIVA foci in red; cross-walls in yellow.
(GIF)

Video S5 Large-scale example of the full model simulation output with 25-fold overexpression of DivIVA. Simulation lasts for about seven hours with overexpression occurring after 14,000 s. Hyphae in green; DivIVA foci in red; cross-walls in yellow.
(GIF)
