# Supplementary material for: Mechanistic Basis of Branch-Site Selection in Filamentous Bacteria
Source: PLoS Comput Biol. 2012 Mar 8;8(3):e1002423. doi: 10.1371/journal.pcbi.1002423 (PMC3297577; doi:10.1371/journal.pcbi.1002423)
Supplement: Text S1 — Supporting text. (PDF) [file pcbi.1002423.s011.pdf]

Supporting Text S1

Data analysis

Tip growth speed

New branches do not grow at the same speed as established branches. Instead the tips of new branches initially extend more slowly and gradually increase in speed before attaining full speed. To quantitatively analyse this increase, we used our time-lapse imaging to measure the extension rate of 45 established and 40 new branches. Figure S1 shows the mean new branch growth speed against time (starting from when the branch first appears), and compares this to the mean growth speed of established hyphae. Using the same data we can also estimate the fluctuations in the initial and established extension speeds, from which we conclude that new branches initially grow at about $v_0 = 4 \pm 2\mu\mathrm{m\,hr}^{-1}$, and then gradually increase (approximately linearly) in speed until they reach $v_{\max} = 8 \pm 4\mu\mathrm{m\,hr}^{-1}$ after about $T = 1.5$ hours.

Subtraction of branch lengths

As explained in *Materials and Methods*, the experimental data do not show new branches at the exact moment that they emerge. Instead it is necessary to infer the tip-to-branch distance, L, at the moment of branching. This involves knowledge of the tip growth speed of new branches, v_0, the tip growth speed of established branches, $v_{\max}$, and how long new branches take to reach full speed, T. However, these three parameters will differ from branch to branch. If fixed values are used then this will lead to incorrect tip-to-branch distances; in extreme cases, this can even lead to negative distances for tip-to-branch distances. Ideally it would be necessary to determine v_0, $v_{\max}$ and T for each branch, although this is not possible from still images. Instead, we determine a distribution of tip-to-branch distances for each measured branch. To do this we allow all three parameters to fluctuate according to Gaussian distributions (which are truncated to ensure $0 < v_0 < v_{\max}$ and $T > 0$). Each set $\{v_0, v_{\max}, T\}$ leads to a tip-to-branch distance and the variations in the parameters leads to a distribution for L. Negative values of L are unphysical and so the distributions are truncated to remove negative distances and rescaled so that they still have unit area. The complete measured tip-to-branch distribution is obtained by summing the distributions derived from all the individual measured branches. The means and standard deviations for v_0, $v_{\max}$ and T are taken from the above data in Oxoid antibiotic

medium. Although these values are likely to be altered in YEME medium, we have tried a wide range of values for each and discovered that changing the values of some or all of v_0, $v_{\max}$ and T by 100% or more makes little difference to the final histogram. Although this may appear counter-intuitive, the absolute tip growth speed cancels out of the branch-subtraction procedure; it is only the difference between v_0 and $v_{\max}$ over the relatively short period T that is relevant.

Hyphal-base to first-offshoot distribution

In order to constrain the value of N_{split}, we measured the distance between the base of hyphae (where the hypha originates from its parent hypha) and the first (i.e. nearest) offshoot branch. If $N_{\text{split}} > \langle N_{\text{br}} \rangle$ then there should be a gap, during which the tip-focus of the new branch is growing in size, before it can form its own offshoot branches. As with measuring the tip-to-branch and branch-to-branch distributions, it is important to impose a trimming protocol. The results at 35μm trim are shown in Figure S2. The data is well fit by a decaying exponential. This is the behaviour expected if N_{split} is equal to (or less than) $\langle N_{\text{br}} \rangle$ since then new tip-foci have the potential to split almost straight away after branching initiation. Since there is no evidence for a gap before new hyphae can form their own branches, we conclude that $N_{\text{split}} \leq \langle N_{\text{br}} \rangle$.

Model robustness

Robustness to changes in mean parameter values and in size of fluctuations

Our model is robust to changes in all eight parameters in Table 1. For example, if we take $\langle N_0 \rangle$ as $3,000$ rather than $1,700$, then, although the distributions and their averages are changed to some extent, there is no overall qualitative difference (Figure S3). The same applies if we decrease $\langle N_0 \rangle$, or if we vary the other parameters by up to 30% of their size.

The minimal model only considers fluctuations in N_0 and N_{br}, which are sufficient to capture the observed distributions. However, there is no reason why the other parameters, in particular the tip growth speed, v, and the on-rate parameter, β, should not also vary. If these are also allowed to vary, even by up to 25% each, then there is no qualitative difference in either the tip-to-branch or branch-to-branch distributions.

Robustness to distribution of fluctuations

In the simplest version of the model, we assume a truncated normal distribution for N_0 with mean $\langle N_0 \rangle = 1,700$ and standard deviation $\delta N_0 = 1,000$. Although simple, this leads to a large, potentially unrealistic, weight for producing foci of very small size. To rectify this it is possible to consider other distributions where the distribution drops towards zero for small initial foci sizes. We considered three types of distribution: log-normal, gamma, and a distribution that is triangular for small foci and Gaussian for large foci. Each distribution had a similar mean and standard deviation to the original truncated Gaussian distribution. In each case there was little qualitative difference from the truncated Gaussian case, showing that the exact shape of the N_0 distribution is not important for our results. We also considered log-normal and gamma distributions for N_{br}, which again made little difference.

Robustness to foci growth dynamics and foci evaporation

In the main text we implemented a rule where a focus containing N DivIVA molecules increases in size at a rate proportional to its size: $\dot{N} = \beta N$. However, we can consider other rules, such as a constant on-rate ($\dot{N} = \beta_0$), or even some combination of the two ($\dot{N} = \beta_0 + \beta N$). Also, we have assumed that foci can capture DivIVA molecules from the cytoplasm but can never return them, i.e. there is no off-rate. However, if we assume that the off-rate is either constant, linear in N, or some combination of the two, then including an off-rate just implies that β_0 and β are rescaled. In any case, we find that these alternative growth laws do not qualitatively change any of our results, and do not lead to a better fit with the experimental data. For example, Figure S4 shows the distributions when a constant growth rule ($\dot{N} = \beta_0$) is implemented.

It is possible that foci can spontaneously evaporate by detaching into the cytoplasm. However, it is difficult to directly observe this potential effect since foci often move out of the focal plane, thereby disappearing. We considered a simple extension to the minimal model where developing foci (i.e. those which have not yet initiated a branch) have a fixed probability per second of evaporating. Even with a probability such that over half of all foci evaporate before initiating a branch, there is little change to the model distributions. This is because the tip-to-branch distribution is determined only by those foci which eventually initiate branches, whereas any change in the branch-to-branch distribution can be compensated by increasing the tip-focus splitting parameter, γ.

Analytic results

Analytic expression for the tip-to-branch distribution

Starting from Eq. (1) and by varying both N_0 and $N_{\rm br}$, we can derive an analytic expression for the distribution of the tip-to-branch distance, L. We assume that both N_0 and $N_{\rm br}$ follow independent truncated normal distributions with means μ_0 and $\mu_{\rm br}$ and standard deviations σ_0 and $\sigma_{\rm br}$ respectively[1]. The probability density function (pdf) for N_0 is given by

$$f_0(N_0) = \begin{cases} 0 & \text{if } N_0 \le 0, \\ \frac{1}{\sqrt{2\pi}\sigma_0 \Phi\left(\frac{\mu_0}{\sigma_0}\right)} e^{-\frac{(N_0-\mu_0)^2}{2\sigma_0^2}} & \text{if } N_0 > 0, \end{cases} \tag{S1}$$

where $\Phi(x)$ is the standard normal cumulative distribution function,

$$\Phi(x) = \frac{1}{\sqrt{2\pi}} \int_{-\infty}^{x} e^{-\frac{1}{2}t^2} dt.$$

A similar expression holds for $f_{\rm br}(N_{\rm br})$, the pdf for $N_{\rm br}$. First, we determine the distribution of $u \equiv \frac{N_{\rm br}}{N_0}$, which we write as $g(u)$. The ratio of two distributions is a standard result:

$$g(u) = \int_{-\infty}^{\infty} |x| f_0(x) f_{\rm br}(ux) dx.$$

Since f_0 and $f_{\rm br}$ vanish for negative values, the lower limit can be replaced by 0 and the $|x|$ by x. Evaluating the integral gives

$$g(u) = \begin{cases} 0 & \text{if } u \le 0, \\ \frac{e^{-\frac{c}{2}}}{\sqrt{2\pi}\sigma_0\sigma_{\rm br}\Phi\left(\frac{\mu_0}{\sigma_0}\right)\Phi\left(\frac{\mu_{\rm br}}{\sigma_{\rm br}}\right)\tilde{a}(u)^{\frac{3}{2}}} \left(\sqrt{\frac{\tilde{a}(u)}{2\pi}} + \tilde{b}(u) e^{\frac{\tilde{b}(u)^2}{2\tilde{a}(u)}} \Phi\left(\frac{\tilde{b}(u)}{\sqrt{\tilde{a}(u)}}\right) \right) & \text{if } u > 0, \end{cases}$$

where

$$\tilde{a}(u) = \frac{1}{\sigma_0^2} + \frac{1}{\sigma_{\rm br}^2} u^2,$$
$$\tilde{b}(u) = \frac{\mu_0}{\sigma_0^2} + \frac{\mu_{\rm br}}{\sigma_{\rm br}^2} u,$$
$$c = \frac{\mu_0^2}{\sigma_0^2} + \frac{\mu_{\rm br}^2}{\sigma_{\rm br}^2}.$$

[1] Here μ_0 and $\mu_{\rm br}$ are the means of the full Gaussians, rather than those of the truncated Gaussians. The same also applies to the standard deviations, σ_0 and $\sigma_{\rm br}$.

Finally, we can determine the distribution of L, $h(L)$, by using $L = \frac{v}{\beta}\ln u$ and the fact that $|h(L)dL| = |g(u)du|$. Negative values of L imply that $N_0 > N_{\rm br}$ and so, as discussed in the main text, these branches will emerge at zero distance from the tip and so should really contribute at $L = 0$. So the entire weight of $h(L)$ for negative L should be placed at $L = 0$. We achieve this by using a delta function at the origin of u. Then our final expression for the tip-to-branch distribution, $\bar{h}(L)$, becomes

$$\bar{h}(L) = \begin{cases} 0 & \text{if } L < 0, \\ \delta(L)\int_{-\infty}^{0} h(\tilde{L})d\tilde{L} + h(L) & \text{if } L \geq 0, \end{cases} \tag{S2}$$

where

$$h(L) = \frac{\beta e^{\frac{\beta}{v}L - \frac{c}{2}}}{\sqrt{2\pi} v \sigma_0 \sigma_{\rm br} \Phi\left(\frac{\mu_0}{\sigma_0}\right)\Phi\left(\frac{\mu_{\rm br}}{\sigma_{\rm br}}\right) a(L)^{\frac{3}{2}}}\left(\sqrt{\frac{a(L)}{2\pi}} + b(L)e^{\frac{b(L)^2}{2a(L)}}\Phi\left(\frac{b(L)}{\sqrt{a(L)}}\right)\right),$$

and where

$$a(L) = \frac{1}{\sigma_0^2} + \frac{1}{\sigma_{\rm br}^2}e^{2\frac{\beta}{v}L},$$
$$b(L) = \frac{\mu_0}{\sigma_0^2} + \frac{\mu_{\rm br}}{\sigma_{\rm br}^2}e^{\frac{\beta}{v}L}.$$

To compare this analytic solution to the numerical simulations, we must convert $\langle N_0\rangle$, $\langle N_{\rm br}\rangle$, δN_0 and $\delta N_{\rm br}$ (the means and standard deviations of the truncated Gaussians) to μ_0, $\mu_{\rm br}$, σ_0 and $\sigma_{\rm br}$ (the means and standard deviations of the full Gaussians). Using the values in Table 1, where $\langle N_0\rangle = 1,700$, $\langle N_{\rm br}\rangle = 10,000$, $\delta N_0 = 1,000$ and $\delta N_{\rm br} = 2,600$, we find that $\mu_0 \approx 1,500$, $\mu_{\rm br} \approx 10,000$, $\sigma_0 \approx 1,200$ and $\sigma_{\rm br} \approx 2,600$.

The resulting distribution (Figure S5) can never be measured experimentally since it corresponds to measuring tip-to-branch distances at infinite trim. However, it is in many ways the "true" underlying distribution, a distribution which is unbiased by experimental limitations.

Analytic expression for the trimmed tip-to-branch distribution

To compare Eq. (S2) with the measured data we must impose the same trimming protocol. By trimming all branches to some trim length Λ, it becomes less likely that we observe branches with longer tip-to-branch distances. This is because such branches emerge from foci which take longer to develop into branches and thus the associated tip-focus splitting event has a smaller time

frame in which it must have occurred. This is illustrated in Figure S6, where a branch with tip-to-branch distance L will only be measured if it was created within $\Lambda - L$ of the base of the hypha; if it is created nearer the tip than this, then the focus will not have originated a new branch by the time it is measured. Thus, assuming a constant probability per unit time of tip-focus splitting (which will be true when a sufficiently large number of hyphae are analysed), the probability of observing such a branch is scaled by a factor of $\Lambda - L$. This implies that the probability density function in Eq. (S2) should be scaled by the same factor. This gives the Λ-trimmed tip-to-branch distribution, $\bar{h}_\Lambda(L)$, as

$$\bar{h}_\Lambda(L) = \begin{cases} 0 & \text{if } L < 0, \\ \left(\frac{\Lambda - L}{\Lambda - \mu_{\bar{h}}}\right) \bar{h}(L) & \text{if } 0 \leq L < \Lambda, \\ 0 & \text{if } L \geq \Lambda, \end{cases} \tag{S3}$$

where $\mu_{\bar{h}} = \int_0^\infty L\bar{h}(L)dL$ is the mean of $\bar{h}(L)$, and the $\Lambda - \mu_{\bar{h}}$ denominator is required to fix the normalisation.

The tip-to-branch distance as a function of the model parameters

In Figure S7 we show how the mode of the tip-to-branch distribution varies with (i) the binding parameter, β, (ii) the mean initial focus size, $\langle N_0 \rangle$, and (iii) the mean focus size for branch initiation, $\langle N_{\text{br}} \rangle$. With an infinite trim the behaviour is given by Eq. (1), which shows that $\langle L \rangle \sim 1/\beta$, $\langle L \rangle \sim \text{const} - \ln\langle N_0 \rangle$ and $\langle L \rangle \sim \text{const} + \ln\langle N_{\text{br}} \rangle$, where $\langle L \rangle$ here represents the mode of L. However, the behaviour is less intuitive when the trimming protocol is imposed. The most interesting case is when β is varied. At large values of β, the modal trimmed tip-to-branch distance tends to the untrimmed value. However, as β is reduced, the trimmed modal value reaches a maximum and begins to drop to zero as β is further reduced. This counter-intuitive behaviour is related to the trim length being much smaller than the true (i.e. infinite trim) modal tip-to-branch distance. It is worth recalling that it is only possible to directly measure the trimmed distribution and so, for any measured trimmed modal tip-to-branch distance, there are two possible values of β. However, it is easy to distinguish the correct value by the number of discarded hyphae (due to imposing the trimming protocol): the smaller β corresponds to a much greater true (i.e. infinite trim) modal distance and so results in a far greater number of discarded hyphae. We do not observe such a large number of discarded hyphae and so our wild-type β is the larger of the two possible values.

The full model

Despite the success of the minimal model described above and in the main paper, it is nevertheless useful to develop a full model including effects such as spatial and temporal gradients of the DivIVA concentration. This is important for two reasons: firstly, it justifies our claims that the extra parts of the full model play only a minor role, and secondly, the full model includes spontaneous nucleation which we need to understand heavy DivIVA overexpression.

Basic components

The full model is a one-dimensional simulation of an entire *Streptomyces* colony. Although there are stochastic elements, the diffusion, production and degradation of DivIVA is handled deterministically (see Table S1 for parameter values). This is justified since DivIVA for our parameters is present at high copy number (hundreds of copies per micron). Each hypha is represented by a 1D array specifying the cytoplasmic DivIVA density at that position, with a focus at the tip. Each site may or may not contain a focus on either the adjacent upper or lower membrane. After a new branch develops, an additional 1D array representing the new branch is generated. At each lattice site and time step, DivIVA is produced, degraded and diffuses using an Euler discretisation of the corresponding partial differential equation, with lattice spacing of $\Delta x = 0.1\mu$m and a time step of $\Delta t = 10^{-4}$s. Diffusion is entirely one-dimensional apart from at points where branches meet, where two-dimensional diffusion occurs. Also, if there is an adjacent focus on the membrane, then DivIVA molecules can be recruited from the cytoplasm to the focus (and also in principle detach from the focus back to the cytoplasm). The number of molecules being recruited to a focus is linearly dependent on both the cytoplasmic DivIVA density at that point, ρ, and the number of molecules in the focus, N, such that $\Delta N = \tilde{\beta}\rho N \Delta t$, where $\tilde{\beta}$ is the binding constant. At each time step, the tip of each branch is extended by $v\Delta t$. Whenever the branch length (as measured in lattice steps) increases through an integer value, an extra lattice site is inserted (with the tip-focus now being adjacent to the newly-inserted site). Furthermore, tip-foci which contain more than N_{split} molecules have a constant probability at each time step of splitting to create new foci, which are placed on the membrane adjacent to the neighbouring cytoplasmic lattice site. When they do so the size of the focus left behind, N_0, is chosen from a truncated Gaussian distribution of the form given in Eq. (S1). At the same time, the size that a focus needs to reach before a new branch is initiated, N_{br}, is chosen from a second truncated

Gaussian distribution of the same form. When that focus finally grows to a size $N_{\rm br}$, a new branch is formed with the focus now sitting adjacent to the cytoplasmic site at the tip of that branch.

Additional processes

To the above form of the model we added various other effects. Firstly, spontaneous nucleation was included, where new foci could now arise at any membrane site along any hypha. This was implemented as a stochastic process where the probability of nucleation per unit time, η, on a membrane adjacent to each lattice site is dependent on the cytoplasmic DivIVA density, ρ, at that adjacent site and on a threshold concentration, $\rho_{\rm SN}$ (see [16]):

$$\eta = \begin{cases} 0 & \text{if } \rho \leq \rho_{\rm SN}, \\ \tilde{\eta}(\rho - \rho_{\rm SN}) & \text{if } \rho > \rho_{\rm SN}, \end{cases} \tag{S4}$$

where $\tilde{\eta}$ is a constant that is independent of ρ. Below the threshold, nucleation is assumed not to occur, whereas, above the threshold, the nucleation probability per unit time is assumed to increase linearly with the DivIVA concentration above the threshold. After nucleation, foci begin with a fixed size of $N_0 = 5$ and with $N_{\rm br}$ chosen in the same way as before, with the DivIVA for the new focus taken from the lattice site directly adjacent to the new focus. Parameter values for this and the other processes discussed here are listed in Table S1. Secondly, we included cross-walls which sometimes appear during vegetative growth and which can be visualised by fluorescently tagging FtsZ [S1]. For our purposes, the main effect of FtsZ is to isolate different compartments, preventing DivIVA from diffusing between them. It was shown in [S2] that FtsZ rings tend to form in a progressive manner, with subsequent Z-rings appearing closer to the tip. Rather than modelling the detailed dynamics of FtsZ and the formation of cross-walls, for each branch we simply included a constant probability per unit time ($1 \times 10^{-4}{\rm s}^{-1}$) of forming a cross-wall; if a cross-wall is formed then its position is chosen randomly between the previous cross-wall and the tip. Thirdly, new branches initially extended at only half the speed of established branches, as found experimentally, thereafter gradually increasing in speed in a linear fashion, to achieve full speed after ninety minutes. Previously, in the minimal model, this effect was included only in the experimental extraction of tip-to-branch distances, rather than in the simulation itself.

Curved branch growth

We next consider non-straight tip-growth and allow the tip-growth direction to vary. It is possible that the curvature of the membrane just next to the tip is a factor influencing when tip-focus splitting occurs. Rather than trying to understand the details of what controls the tip-growth direction (not currently a tractable problem), at each time step we simply choose the new growth direction as the previous growth direction plus a Gaussian-distributed correction with zero mean. The width of this Gaussian ($3.5°$ per new lattice site) is determined by the persistence length (1.6μm), which is the distance over which correlations in the growth direction are maintained. Once curved tip-growth is implemented, we can replace the tip-focus splitting parameter with a rule based on curvature: since DivIVA may preferentially form foci on negatively-curved membranes, we implement a rule where tip-foci split only if the local curvature near the tip (the change in tip direction over the last 1μm of growth) is sufficiently high (greater than $15°$). This curvature threshold is chosen to reproduce the tip-focus splitting probability per unit time and to correctly match the branch-to-branch distribution. We also allow for a small probability of focus deposition on the membrane with the "wrong" local curvature (positive rather than negative; see Table S1).

Results

The full model (which uses the parameters in Table S1) produces output such as Videos S3 and S4, which match well with the observed *Streptomyces* phenotypes both in the wild type and when DivIVA is overexpressed. Despite the addition of effects such as cross-walls, DivIVA gradients and curvature-dependent tip-focus splitting, the full model is practically indistinguishable from the minimal model. In particular, there is no significant change in the tip-to-branch or branch-to-branch distributions. Thus the minimal model outlined in the main paper is sufficient to capture branching dynamics in *Streptomyces*. The full model is only needed when spontaneous nucleation becomes an important effect, such as when DivIVA is heavily overexpressed.

Table S1: Additional model parameters and their values

Parameter	Value
DivIVA cytoplasmic diffusion constant, D	$5\mu\text{m}^2\text{s}^{-1}$
DivIVA cytoplasmic production, μ	$0.2\mu\text{m}^{-1}\text{s}^{-1}$
DivIVA cytoplasmic degradation rate, ν	$5 \times 10^{-4}\text{s}^{-1}$
Binding constant, $\tilde{\beta}$	$3 \times 10^{-7}\mu\text{ms}^{-1}$
Spontaneous nucleation threshold, ρ_{SN}	$400\mu\text{m}^{-1}$
Spontaneous nucleation parameter, $\tilde{\eta}$	$5 \times 10^{-8}\mu\text{ms}^{-1}$
FtsZ ring creation probability per unit time	$1 \times 10^{-4}\text{s}^{-1}$
Distribution width for new growth direction	3.5°
Local curvature length	$1\mu\text{m}$
Tip-focus splitting curvature threshold	15°
Probability of "wrong"-side splitting	0.05

Supporting Text S1 References

[S1] Grantcharova N, Lustig U, Flärdh K (2005) Dynamics of FtsZ Assembly during Sporulation in *Streptomyces coelicolor* A3(2) *J. Bacteriol.* 187:3227-3237.

[S2] Jyothikumar V, Tilley EJ, Wali R, Herron PR (2008) Time-lapse microscopy of *Streptomyces coelicolor* growth and sporulation. *Appl. Environ. Microbiol.* 74:6774-6781.

Supporting Figure Legends

Figure S1: Tip growth speed against time in Oxoid antibiotic medium for an established hypha and a newly formed branch. Error bars show the standard error of the mean.

Figure S2: Experimental distribution of distances from parent hypha to first offshoot at 35μm trim. 44 data points.

Figure S3: Comparison of model histograms at 80μm trim with $\langle N_0 \rangle = 1,700$ and $\langle N_0 \rangle = 3,000$. (A) Tip-to-branch distribution. (B) Branch-to-branch distribution.

Figure S4: Comparison of histograms at 80μm trim for linear growth model ($\dot{N} = \beta N$, parameters in Table 1) and constant growth model ($\dot{N} = \beta_0$, $v = 8\mu\mathrm{m hr}^{-1}$, $\beta_0 = 0.29\mathrm{s}^{-1}$, $\langle N_0 \rangle = 1,300$, $\delta N_0 = 850$, $\langle N_{\mathrm{br}} \rangle = 10,000$, $\delta N_{\mathrm{br}} = 3,000$, $\gamma = 2.5 \times 10^{-3}\mathrm{s}^{-1}$, $N_{\mathrm{split}} = 10,000$). (A) Tip-to-branch distribution. (B) Branch-to-branch distribution.

Figure S5: Analytic tip-to-branch distribution with infinite trim. This represents the "true" underlying distribution which can never be directly measured experimentally.

Figure S6: Requirement for a branch to be included in the data set. (A) A growing branch which will be measured when it has grown another $\Lambda\mu$m. (B) A new focus is created at distance x from the base. (C) This focus develops into a branch after the tip has grown a further $L\mu$m, i.e. this branch has a tip-to-branch distance of $L\mu$m. (D) Only branches within Λ of the tip are used to collect data. So this branch will only be recorded if $x + L < \Lambda$.

Figure S7: Behaviour of the mode of the tip-to-branch distance distribution as a function of various model parameters, for both an infinite trim (blue line) and an 80μm trim (red line). The infinite trim line is always higher than the 80μm trim line. The black dotted line shows the wild-type parameter value. (A) As a function of the binding parameter, β. (B) As a function of the mean initial focus size, $\langle N_0 \rangle$. (C) As a function of the mean focus size for branch initiation, $\langle N_{\text{br}} \rangle$.

Figure S8: Comparison of distributions between the minimal model and experimental data at 60μm trim. Analytic tip-to-branch distribution is also shown (curved line). (A) Tip-to-branch distribution. 1876 experimental data points. (B) Zoomed tip-to-branch distribution. (C) Branch-to-branch distribution. 1215 experimental data points.

Figure S9: Comparison of distributions between the minimal model and experimental data at 100μm trim. Analytic tip-to-branch distribution is also shown (curved line). (A) Tip-to-branch distribution. 297 experimental data points. (B) Zoomed tip-to-branch distribution. (C) Branch-to-branch distribution. 257 experimental data points.

Figure S10: Schematic of colony morphology for various values of the binding parameter, β. Red dots represent DivIVA foci. (A) Small value of β. (B) Wild-type value of β. (C) Large value of β.

Video Legends

Video S1: Movie version of Figure 1. Evidence of tip-focus splitting, growth of foci and emergence of branches, in fluorescence-imaged *Streptomyces coelicolor* expressing *divIVA-egfp*.

Video S2: Movie version of Figure 3. Example of branching at almost zero distance from the tip.

Video S3: Example of the full model simulation output, showing *Streptomyces* starting from a spore and growing for about fourteen hours. Hyphae in green; DivIVA foci in red.

Video S4: Large-scale example of the full model simulation output, showing *Streptomyces* starting from a spore and growing for about eleven hours. Hyphae in green; DivIVA foci in red; cross-walls in yellow.

Video S5: Large-scale example of the full model simulation output with 25-fold overexpression of DivIVA. Simulation lasts for about seven hours with overexpression occurring after 14,000s. Hyphae in green; DivIVA foci in red; cross-walls in yellow.
