# Supplementary figures and images for: Mechanistic Basis of Branch-Site Selection in Filamentous Bacteria
Source: PLoS Comput Biol. 2012 Mar 8;8(3):e1002423. doi: 10.1371/journal.pcbi.1002423 (PMC3297577; doi:10.1371/journal.pcbi.1002423)

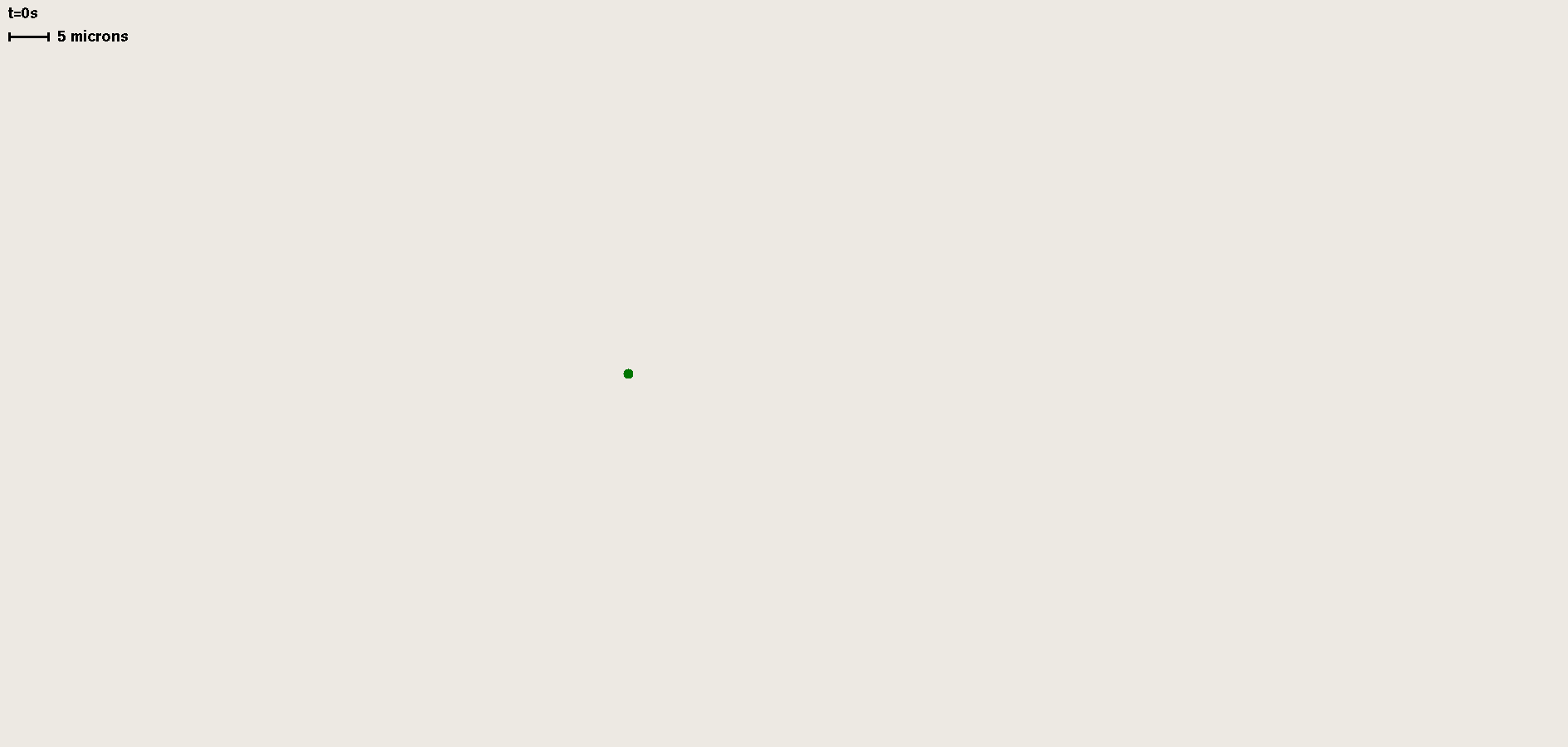

Supplement: Video S3 — Example of the full model simulation output, showing Streptomyces starting from a spore and growing for about fourteen hours. Hyphae in green; DivIVA foci in red. (GIF) [file pcbi.1002423.s014.gif]

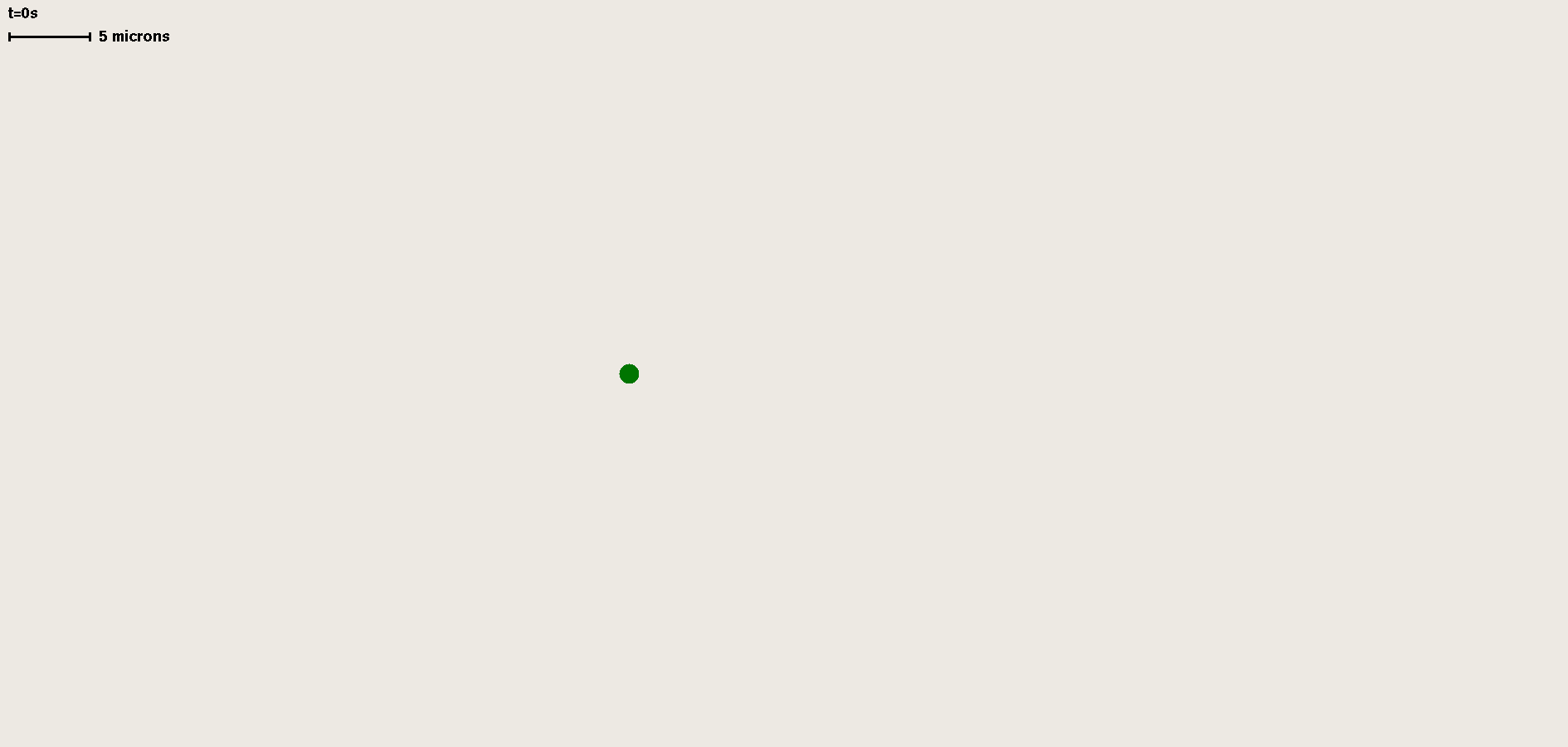

Supplement: Video S4 — Large-scale example of the full model simulation output, showing Streptomyces starting from a spore and growing for about eleven hours. Hyphae in green; DivIVA foci in red; cross-walls in yellow. (GIF) [file pcbi.1002423.s015.gif]

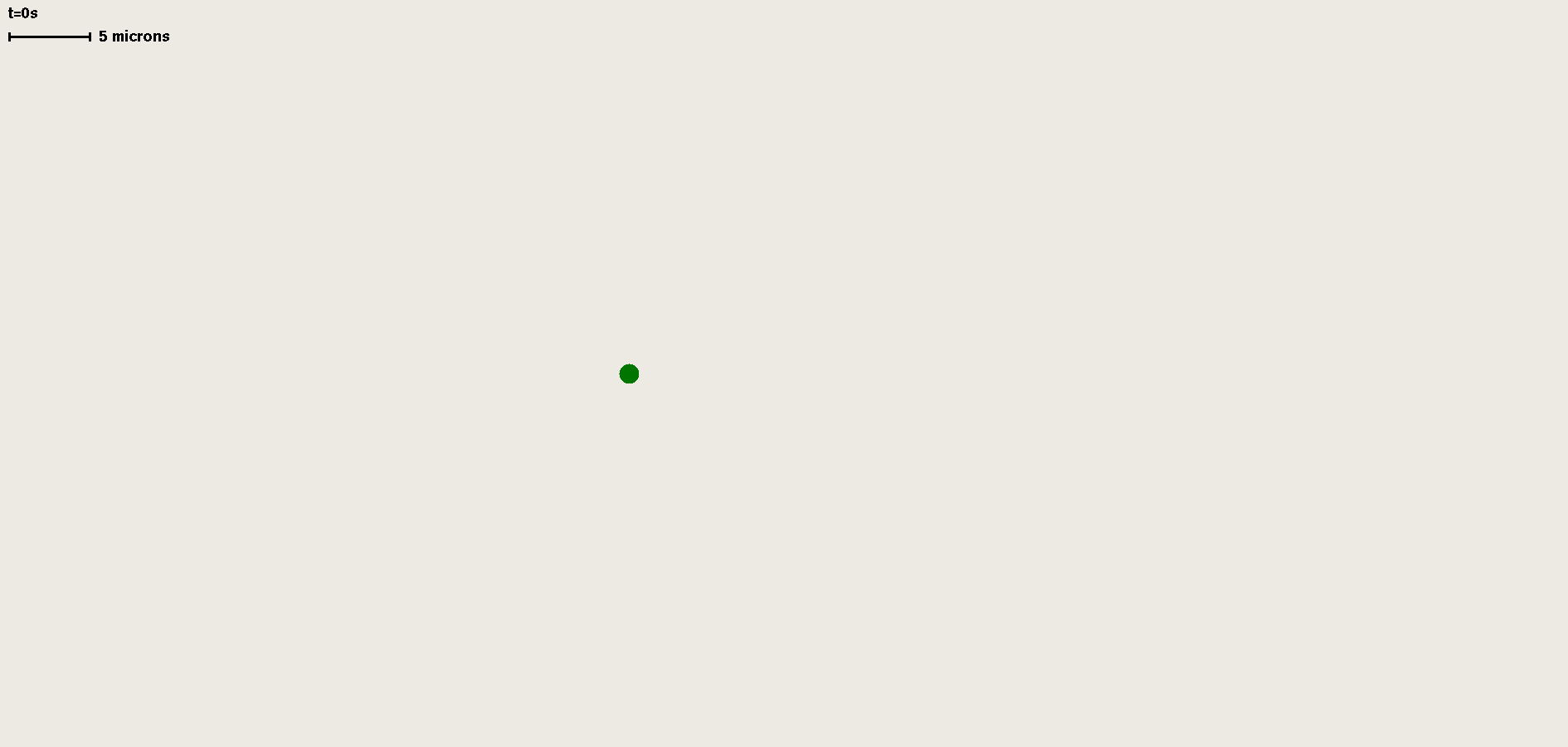

Supplement: Video S5 — Large-scale example of the full model simulation output with 25-fold overexpression of DivIVA. Simulation lasts for about seven hours with overexpression occurring after 14,000 s. Hyphae in green; DivIVA foci in red; cross-walls in yellow. (GIF) [file pcbi.1002423.s016.gif]
